# The effect of insecticide-treated bed nets on the incidence and prevalence of malaria in children in an area of unstable seasonal transmission in western Myanmar

**DOI:** 10.1186/1475-2875-12-363

**Published:** 2013-10-11

**Authors:** Frank M Smithuis, Moe Kyaw Kyaw, U Ohn Phe, Ingrid van der Broek, Nina Katterman, Colin Rogers, Patrick Almeida, Piet A Kager, Kasia Stepniewska, Yoel Lubell, Julie A Simpson, Nicholas J White

**Affiliations:** 1 Medical Action Myanmar, Kokkine Swimming Club Lane 32A1, Yangon, Myanmar; 2 Médécins sans Frontières Holland, Thanlin Road 62A, Yangon, Myanmar; 3 Centre for Infectious Diseases, Tropical Medicine & AIDS, Academic Medical Center, Amsterdam, The Netherlands; 4 Mahidol-Oxford Tropical Medicine Research Unit (MORU), Faculty of Tropical Medicine, Mahidol University, 3rd Floor, 60th Anniversary Chalermprakiat Building, 420/6 Rajvithi Rd., Ratchathewi District, Bangkok 10400, Thailand; 5 Centre for Molecular, Environmental, Genetic & Analytic Epidemiology, Melbourne School of Population and Global Health, The University of Melbourne, Melbourne, Australia; 6 Centre for Tropical Medicine, CCVTM, Churchill Hospital, Oxford, UK

**Keywords:** Malaria, *P. falciparum*, *P. vivax*, Insecticide treated bed nets, Entomology, Biting time, Myanmar, Cost-effectiveness, Cluster randomized controlled trial

## Abstract

**Background:**

Insecticide-treated bed nets (ITN) reduce malaria morbidity and mortality consistently in Africa, but their benefits have been less consistent in Asia. This study’s objective was to evaluate the malaria protective efficacy of village-wide usage of ITN in Western Myanmar and estimate the cost-effectiveness of ITN compared with extending early diagnosis and treatment services.

**Methods:**

A cluster-randomized controlled trial was conducted in Rakhine State to assess the efficacy of ITNs in preventing malaria and anaemia in children and their secondary effects on nutrition and development. The data were aggregated for each village to obtain cluster-level infection rates. In total 8,175 children under 10 years of age were followed up for 10 months, which included the main malaria transmission period. The incidence and prevalence of *Plasmodium falciparum* and *Plasmodium vivax* infections, and the biting behaviour of *Anopheles* mosquitoes in the area were studied concurrently. The trial data along with costs for current recommended treatment practices were modelled to estimate the cost-effectiveness of ITNs compared with, or in addition to extending the coverage of early diagnosis and treatment services.

**Results:**

In aggregate*,* malaria infections, spleen rates, haemoglobin concentrations, and weight for height, did not differ significantly during the study period between villages with and without ITNs, with a weighted mean difference of −2.6 *P. falciparum* episodes per 1,000 weeks at risk (95% Confidence Interval −7 to 1.8). In areas with a higher incidence of malaria there was some evidence ITN protective efficacy. The economic analysis indicated that, despite the uncertainty and variability in their protective efficacy in the different study sites, ITN could still be cost-effective, but not if they displaced funding for early diagnosis and effective treatment which is substantially more cost-effective.

**Conclusion:**

In Western Myanmar deployment of ITNs did not provide consistent protection against malaria in children living in malaria endemic villages. Early diagnosis and effective treatment is a more cost effective malaria control strategy than deployment of ITNs in this area where the main vector bites early in the evening, often before people are protected by an ITN.

## Background

Malaria is a major cause of morbidity and mortality in Myanmar. Malaria control activities in this country have been concentrated on early, mostly clinical, diagnosis and treatment. Limited availability of curative services in remote areas, the difficulties in accessing malarious areas, and the relatively high costs of effective treatment of multi-drug resistant malaria, have compromized malaria control efforts. Effective malaria control activities are needed, and there is a recent substantial increase in donor support for these activities [1]. Levels of chloroquine and sulphadoxine-pyrimethamine resistance in *Plasmodium falciparum* have been high in this region for decades [2,3]. Subsidies for highly effective artemisinin combination treatment (ACT) [4,5] have considerably increased their availability in recent years. Unfortunately artemisinin monotherapies are still widely available providing continued selective pressure on emerging artemisinin resistance in the east of the country [6,7]. Regular and systematic indoor spraying of residual insecticides was stopped in 1993 and is now only used for special situations (outbreaks, new settlements).

Largely ignored by the outside world until 2007, since then there has been an over fifty-fold increase in external donor funding for malaria control in Myanmar, the majority of which has been spent on insecticide-treated mosquito nets (ITN). ITNs are an appealing approach to the control of malaria. They repel and kill malaria vectors, and they prevent sporozoite-bearing anopheline mosquitoes from biting the occupants lying under or near the bed net. ITNs have been shown to exert a mass effect reducing vector populations in villages with extensive use and high transmission [8]. ITNs are usually greatly appreciated by their occupants, because they prevent all kinds of insect bites, and they are very safe. Overall use of ITNs prevented approximately one in five childhood deaths in sub-Saharan Africa [9]. These substantial benefits have rightly led to the inclusion of ITNs as a central component of many malaria control initiatives throughout the world. In endemic areas of South and South-East Asia, where malaria transmission is lower and unstable, mobile young adult males are particularly affected, and vector behaviour is different, the results of ITN efficacy studies are mixed, and much less clear than in Africa [10-14]. This is not because of insecticide resistance, although some vector species do exhibit low-grade pyrethroid resistance [15], but because of the behaviour of the vectors which often bite early in the evening or morning outdoors or away from dwellings, and the corresponding behaviour of humans. ITN always provide some protection against malaria – the critical question is how much? All individuals in malaria endemic areas should ideally have ITN, even if they provide only modest benefit, but whilst there are financial constraints, choices need to be made. It is necessary therefore to assess the efficacy of ITN in the South and South-East Asian region at a local level to assist decisions on their wider-scale deployment in relation to alternative proven highly effective malaria control measures (i.e. early diagnosis and effective treatment: EDAET). The effectiveness of ITN in reducing malaria morbidity and mortality depends on several factors, including the level of malaria endemicity and the behaviour and immunity of the population, the climate, the acceptance and usage of the ITN by the population and, crucially, the biting behaviour of the main anopheline vectors [16].

A cluster-randomized controlled trial was carried out in Western Myanmar, to evaluate the protective efficacy of village-wide usage of insecticide-impregnated ITNs on malaria incidence and prevalence, anaemia, and the development of children. The anopheline vector abundance and biting behaviour were also studied, and data were collected on population sleeping habits, to assess the potential of ITNs to reduce man-mosquito contact in this region; these findings are described in the accompanying paper.

While effective diagnosis and treatment facilities were an ethical and research necessity in the study setting, these are often not present in routine settings. Budgetary limitations may result in ITN programmes competing with EDAET services for limited resources. A cost-effectiveness analysis was therefore carried out to assess the costs and benefits of ITN when compared with those of EDAET when neither has been implemented, and to assess the incremental cost-effectiveness of introducing one of the interventions when the other is already in place.

## Methods

### Study area and population

**Figure 1:**
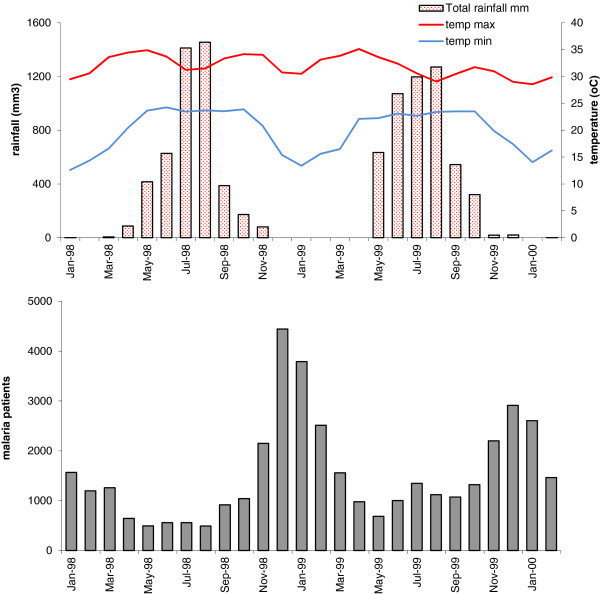
**Climate and seasonal malaria incidence in Rakhine State in 1998–1999. Upper**. Monthly rainfall and minimum/maximum temperature, average of Sittwe and Maungdaw Townships (data obtained from township weather stations). **Lower**. Monthly number of malaria-patients visiting 6 malaria clinics in the region (not only at the study sites).

The study was conducted in Rakhine State, Western Myanmar. The monsoon season is from May to October and yearly rainfall is very high (+/− 5000 mm/year). Malaria transmission occurs throughout the year and peaks during the post monsoon (November-January), and sometimes in the early monsoon (June-July) periods (Figure 1). The transmission intensity varies from predominantly low to pockets of intense transmission, depending on the location, and there are considerable variations over short geographic distances and also between years and between seasons. Symptomatic malaria occurs at all ages but is seen predominantly in children. Access to effective treatment has historically been very poor. Outbreaks of falciparum malaria can spread over several townships, or may be limited to a single village at other times.

In 1994, *Médécins Sans Frontières - Holland* (MSF-H) began a malaria control programme in Rakhine State in cooperation with the Myanmar VBDC (Vector Borne Disease Control) department [2]. The programme focussed on early diagnosis and treatment, through support of 30 fixed field clinics with laboratories and riverboat ‘mobile malaria clinics’. *Plasmodium falciparum* was responsible for approximately 80% of the malaria infections in patients who presented to these clinics. Drug resistance in *P. falciparum* in the area was studied in 1995; high levels of resistance were found with treatment failure rates of 82% to chloroquine and 67% to sulphadoxine-pyrimethamine, whereas mefloquine was very effective (93% cure rate) [2]. Since 1996 all patients with falciparum malaria have been treated with a combination of mefloquine and artesunate [3-5]. Between 1997 and 2007 over one million patients (approximately 43% of whom were children under 10 years of age) received treatment for slide confirmed malaria, supported by this programme. It was unclear whether ITN should be deployed as a priority so the effectiveness of ITN was studied between May 1998 and February 1999, covering the peak malaria transmission season, in villages around two Rural Health Clinics (RHC), in Dabhine and Myothugyi, two village-tracts located in the townships Sittwe and Maungdaw, which are approximately 90 km apart. The study villages in the Dabhine area are all within 5 miles from the Bay of Bengal. This is a coastal plain area without hills or forest, where rice and other crops are cultivated. Common breeding sites for *Anophelines* are paddy fields, brackish water in streams and tide pools and saline ponds used for prawn culture which are close to villages, swamps, and small ponds used for growing watercress. The area was classified generally as meso- to hyperendemic for malaria. A malaria blood-slide survey, performed in September 1995, found a prevalence of 34% for *P. falciparum* and 11% for *Plasmodium vivax,* and a spleen prevalence of 36% among primary school children.

Myothugyi is a densely populated area situated 5–7 miles from the Bay of Bengal. The area is characterized by rice-fields and partly forested hills. Potential breeding sites for *Anophelines* are freshwater creeks, ponds and stagnant water in rice fields, as well as brackish water in tide pools, prawn-breeding ponds and small pools and streams at foothills. It has been classified generally as a low meso-endemic malaria area. A malaria blood-slide survey in January 1996, 10 kilometres south of Myothugyi, found a prevalence of 13% for *P. falciparum* and 3% for *P. vivax* and none of the primary schoolchildren examined had palpable splenomegaly. These surveys, and the substantial burden of malaria in children, prompted discussions with the community and authorities as to what control measures could be applied. In view of the uncertainties over ITN efficacy and their limited availability at the time, and after consultation with the authorities and village leaders, it was decided jointly that an evaluation was needed to set priorities before consideration of wide-scale deployment. It was agreed that if there was any evidence of benefit, then after the evaluation, ITNs would be given to all villages included in the evaluation**.**

### Study design, randomization and sample size calculation

Ideally, a study population is randomized on an individual or household basis. However, the use of insecticide-impregnated bed nets (ITN) has an impact on the vector populations in the nearby environment and, therefore, on malaria transmission and risk, not only in the household itself, but also among people living nearby, including those who do not have ITN (the “mass” effect). Therefore, the unit chosen for randomization in this study was the village [17], and this was agreed with the village leaders in consultation with the community. The primary outcome measure was the incidence and prevalence of malaria in children.

In December 1997 and January 1998 a population survey was done in the study areas. Outreach Workers (ORW) were trained to register all families, number houses, and to collect demographic data on family size and number of children under 10 years of age. During this period, a sample of the population of children under 10 was checked for parasitaemia and splenomegaly. The results of the pre-intervention survey were used to determine the number of clusters and the sample sizes needed for the larger study.

The pre-intervention malaria survey was performed on 1,088 children (Table 1). The intra-class correlation coefficient (ICC) calculated on the basis of the pre-intervention data was 0.048, with a between-cluster variation of 0.006 and within-cluster variation of 0.12 [18]. The prevalence of *P. falciparum* parasitaemia by microscopy averaged 17% (range 0 – 53%). Microscopy was quality assured throughout the study with independent cross-checking. The number of village-clusters and their size needed for a valid comparative study was calculated using the methods described by Thompson [19]. In order to detect a 50% reduction in the incidence of *falciparum* malaria, with 80% power at a 5% significance level, a total of 10 village pairs were required with an average of 420 children per village, amounting to a total sample size of 8,400 children.

**Table 1:** ***Plasmodium falciparum, Plasmodium vivax *and splenomegaly prevalences in ITN and control villages during the pre-study malaria survey (December 1997)**

	**Intervention villages**					**Control villages**				
	**N**	**M.S.**	***Pf *(%)**	***Pv *(%)**	**Spleen (%)**	**N**	**M.S.**	***Pf *(%)**	***Pv *(%)**	**Spleen (%)**
**Dabhine**										
Pair 1	707	125	37 (30)	35 (28)	79 (63)	796	102	38 (37)	33 (32)	68 (67)
Pair 2	551	89	26 (29)	33 (37)	47 (53)	679	48	9 (19)	7 (15)	16 (34)
Pair 3	462	37	8 (22)	10 (27)	16 (42)	298	49	11 (22)	6 (12)	30 (61)
Pair 4	223	33	7 (21)	8 (24)	9 (28)	184	32	4 (13)	16 (50)	19 (59)
Pair 5	74	18	3 (17)	4 (22)	5 (28)	87	15	8 (53)	1 (7)	6 (40)
Subtotal	2017	302	81 (27)	90 (30)	156 (52)	2044	246	70 (28)	63 (26)	139 (57)
**Myothugyi**										
Pair 6	78	16	3 (19)	1 (6)	6 (38)	47	15	3 (20)	0 (0)	1 (7)
Pair 7	714	96	1 (1)	0 (0)	15 (16)	819	105	0 (0)	0 (0)	30 (29)
Pair 8	492	54	0 (0)	1 (2)	13 (24)	602	70	2 (3)	1 (1)	6 (9)
Pair 9	659	57	4 (7)	4 (7)	7 (12)	650	82	1 (1)	0 (0)	1 (1)
Pair 10	153	25	10 (40)	2 (8)	8 (32)	120	20	7 (35)	1 (5)	4 (20)
Subtotal	2096	248	18 (7)	8 (3)	49 (19)	2238	292	13 (4)	2 (1)	42(14)
Total	4113	550	99 (18)	98 (18)	205 (37)	4282	538	83 (15)	65 (12)	181 (34)

N = number of children < 10 yrs (data from local authorities).

MS = number of children of whom a malaria smear was taken.

*Pf* = *P. falciparum* prevalence (including mixed infections), *Pv* = *P. vivax* prevalence (including mixed infections).

Initially, 22 villages were informed about the study-procedures and were invited to participate, but after further discussions two villages declined to join. Finally 20 villages (clusters) were paired, and for each pair one was selected randomly (using a computer generated random number) to receive impregnated bed nets (ITNs), while the other acted as the control village. Matching was done according to geographical location so that the intervention and control villages were nearby (villages were 1–2 miles apart). This minimized differences in the environment in and around each village pair (i.e. proximity to foothills, forest fringe, prawn-ponds, rice-fields, waterways), which could have had a strong influence on vector populations and consequently on the prevalence and incidence of malaria. The travel-time to the health clinic and its influence on the number of patient visits, and therefore on malaria incidence, were comparable for intervention and control villages, as the distances were matched equally.

### ITN distribution

**Figure 2:**
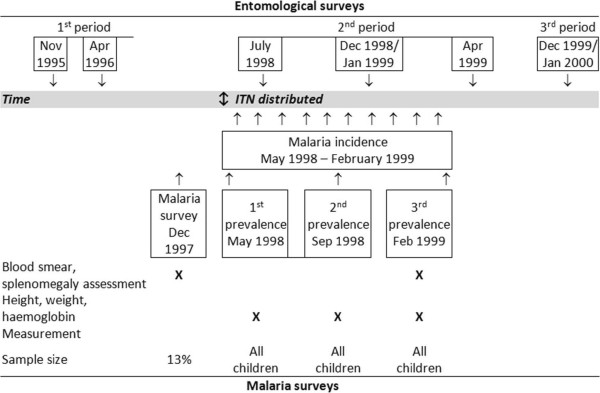
**Time frame of the study of insecticide treated mosquito nets and entomological surveys **[17]** between 1995 and 2000 in the study areas.**

Between the 26th and 29th of April 1998, all households in the intervention villages received a number of ITNs, according to the number of family members and were instructed explicitly about the correct use of the nets (Figure 2). More than 5000 ITN were distributed. The ITN (green colour, polyester, size 130 × 180 × 150 cm (11.6 m^2^) or 190 × 180 × 150 cm (14.5 m^2^), Siam-Dutch Co, Thailand) were already impregnated with deltamethrin (25 mg/m^2^) by the manufacturer. All households of the control villages received ITNs after the study was completed in May 1999.

### Incidence surveillance

Malaria morbidity surveillance consisted of passive case detection. Each family from both study groups received a specific registration card at the beginning of the study and anyone with complaints of fever was urged to visit the RHC and bring the registration card. In both study areas a RHC operated by staff from the Department of Health, was supported by doctors, microscopists, and out-reach workers from MSF-H. Microscopy was quality checked on a regular basis. The RHCs were open seven days a week. Out-reach workers regularly visited the villages and informed the population about using the clinics in case of illness. In the RHC blood-smears of patients with fever or a history of fever were examined for malaria parasites. Data-files were kept for all children under 10 years coming from the villages under study. Patients with falciparum malaria were treated with a single dose of mefloquine 15 mg base/kg and artesunate 4 mg/kg (then the current treatment) [4]. Vivax malaria was treated with chloroquine (10 mg base/kg on day 0 and 1, 5 mg/kg on day 2), followed by primaquine (0.25 mg base/kg/day for 14 days). Patients who were not part of the study were also provided with medical services. No other significant health services were available in the areas under study.

### Cross-sectional surveys

After distribution of ITN, children were followed up for weight, height and haemoglobin at three time-points: at the beginning of the study (May 11 - June 10, 1998), after 5 months (14 Sept- 9 Oct. 1998) and at the end of the study (25 Jan - 22 Feb 1999) (Figure 2). During the last survey a blood-smear for the detection of malarial parasitaemia was taken as well. During the surveys people were asked about the usage of ITN and whether they had washed their ITN.

### Ethics statement

The study was discussed in detail with community representatives of each village and with officials from the Ministry of Health. There was general agreement on the need for an evaluation but no local ethical review committee or relevant formal organizational ethics review process was available before the study started in 1998. The reasons for the study were discussed and it was explained that individual or community decision to participate or not would not in any way jeopardize anti-malarial screening, treatment, or subsequent deployment of ITN. Villages were included in the study only after extensive discussions and full approval of the village representatives. Many study participants were illiterate, and for these individuals fully informed witnessed verbal consent was obtained from adults and the parents of children involved in the study. The Myanmar National Health authorities granted approval for this study.

### Statistical analysis

#### Malariometric data

This study was designed as a cluster randomized trial and, therefore, the data were aggregated for each village (i.e. cluster) to obtain cluster-level infection rates. This approach is appealing because the cluster is both the unit of randomization and the unit of analysis. The villages with bed nets were then compared to the other villages using a weighted paired *t* test [19,20]. The results of blood smears, collected at the clinics, were used to calculate the incidence density of *P. falciparum*, defined as the number of malaria episodes per 1,000 child-weeks at risk. The number of weeks at risk for each child was calculated from the start of the study till the end of the study (the last malaria prevalence survey) or the final date they were seen before they moved or died. If children moved in the first half of the study (before the 2nd prevalence survey), the weeks at risk could be not calculated and these children were, therefore, excluded from the analysis of malaria episodes per 1,000 child weeks at risk. If children moved in the second half of the study, the weeks at risk were taken from the first half of the study. Children who had falciparum malaria were treated with mefloquine and artesunate and were considered not at risk of reinfection because of post-treatment prophylaxis for four weeks following treatment. Three categorical variables were also calculated, in which each child was classified to have one or more infections with either *P. falciparum, P. vivax* or “all species of malaria (i.e. any malaria)” between 11 May 1998 and 27 February 1999. This was analysed on an intention-to-treat basis. Data from the cross-sectional surveys were also aggregated to obtain cluster-level prevalences of malaria, anaemia, and malnutrition.

### Cost-effectiveness analysis

Four scenarios were modelled for the costs and benefits of malaria control using ITN and/or EDAET as compared with a baseline of no intervention. The study data on incidence of *P. falciparum* cases in the individual villages were used, with Poisson distributions assigned to each of these independently, to capture the uncertainty and variability in these findings. Incidence data for vivax cases per village were not available and the proportion of children with at least one case of vivax over the study period was, therefore, adapted as a proxy for incidence (likely to be an under-estimate of actual incidence), and converted to the rate per 1,000 person weeks. The costs of diagnosis, treatment and ITN were adapted to present day (2013) recommended strategies and their current costs (i.e. rapid tests for the diagnosis of malaria prior to treatment, the current recommended dosage of artemether-lumefantrine for falciparum malaria, continued use of chloroquine with primaquine for vivax malaria, and the use of long-lasting insecticide treated nets (LLIN) instead of ITN). A cost for village malaria workers was also included, that would be required for the EDAET strategy at $1.05 per child per annum (adapted from a study of VMWs in Cambodia [21]).

The costs of treatment are based on the currently recommended dose regimen of 3 days of artemether-lumefantrine (current first line policy); the provider unit costs in Myanmar in 2013 for a full treatment course for children weighing between 5-14 kg was $0.47 and for children weighing 15-24 kg it was $0.94. The cost of one chloroquine tablet was $0.021 and for primaquine it was $0.015. The cost of RDTs was estimated at $0.8. An extra 15% was added to the costs of diagnostics and treatment for shipping and wastage. The cost of LLIN including delivery was assumed to be $10 based on a recent review of LLIN costs which conformed with estimates from donors currently active in Myanmar [18]. It was assumed that each ITN is shared by 1.8 people on average (while ITN can be shared by two children a family of five will receive three nets) and that they have a useful life of three years.

These costs were applied for each 1,000 person-weeks. Clinical cases of falciparum malaria were converted to disability-adjusted life-years (DALYs) assuming a mortality rate of 0.1% in treated cases and 1% in untreated *P falciparum* cases, and a mortality rate of 0.01% and 0.05% in vivax malaria respectively. Modelling also assumed a one-week total duration of illness for uncomplicated treated malaria and four weeks for untreated malaria. Results were generated by running 10,000 Monte Carlo simulations sampling from the probability distributions and calculating the mean costs and effects for each strategy. Cost-effectiveness acceptability curves were generated to summarize the parameter uncertainties and identify which strategy was most likely to be cost-effective across a range of willingness to pay thresholds.

## Results

### Characteristics of study groups

During December 1997 and January 1998 data were collected from 1,088 children (13.3% of the total study population) on the prevalence of malaria and palpable spleens. There was considerable variability in the proportions of children with *P. falciparum* and/or *P. vivax* parasitaemia*,* and palpable spleens among the clusters (Table 1). In May 1998, immediately after the ITN were distributed (but before ITN could have had an impact on malaria related pathology), base-line demographic and clinical data of the study children were gathered. A total of 8,175 children were recruited from twenty clusters. The age and sex distributions of children were similar for the intervention (4,066 children) and control villages (4,109 children), as were the variables “weight for height”, and the proportions of children with anaemia (Hgb < 10.0 g/dL).

### Incidence of malaria

**Figure 3:**
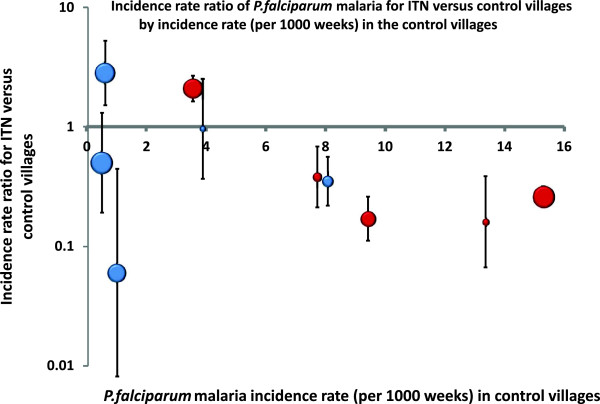
**Incidence rate ratio of *P.falciparum *for ITN versus control (NN) villages by incidence rate (per 1000 weeks) in the control villages.**

During the study-period (10 May 1998 to 28 February 1999) [42 weeks], 2,408 children visited the clinics with complaints of fever and were confirmed by microscopy to have malaria; 1,102 *P. falciparum* (46%), 1,257 *P. vivax* (52%)*,* and 49 mixed i.e. with both species (2%). Of the children infected with *P. falciparum* who visited the clinics, 15.5% came twice with a *P. falciparum* infection (32 from ITN clusters and 117 children from NN clusters), 1.8% came three times (two from ITN cluster and 15 from NN clusters), and three children (NN cluster) came four times with a *P. falciparum* infection during the 10-month follow up period. The number of *P. falciparum* episodes per 1,000 child-weeks at risk was calculated for each cluster (Table 2, Figure 3). There was considerable heterogeneity in the effects. Eight out of the ten ITN villages showed some protective efficacy against falciparum malaria compared to their control village while the remaining two village pairs showed the opposite trend. The study design (by cluster) provides a limited number of data points so further investigation by stratifying clusters by transmission and other characteristics was not feasible. The protective effect of ITN appeared to increase in both relative and absolute terms with the incidence of malaria in the control village in each cluster pair (Figure 3). However the weighted difference between the intervention and control villages for the incidence density of *P. falciparum* was not statistically significant (weighted mean difference of −2.6 episodes per 1,000 weeks at risk, (95% Confidence Interval; -7 to 1.8); p = 0.22).

**Table 2:** ***Falciparum *malaria incidence per 1,000 weeks exposure in ITN and control villages (from 10 May 1998 to 28 February 1999)**

	**ITN villages**			**Control villages**			**ITN Protective efficacy**	
	**N**	***Pf *episodes per 1,000 weeks exposure**		**N**	***Pf *episodes per 1,000 weeks exposure**		**Rate ratio**	**Rate difference**
**Dabhine**								
Pair 1	677	4.04	(97 /24034)	715	15.25	(376 /24662)	0.26	−11.21
Pair 2	522	7.37	(137 /18592)	653	3.51	(83 /23625)	2.09	+3.86
Pair 3	429	1.57	(24 /15304)	280	9.38	(91 /9697)	0.17	−7.81
Pair 4	208	2.83	(21 /7411)	171	8.03	(48 /5980)	0.35	−5.20
Pair 5	67	2.13	(5 /2343)	71	13.32	(33 /2477)	0.16	−11.19
**Myothugyi**								
Pair 6	74	3.68	(9 /2447)	44	3.84	(6 /1562)	0.96	−0.16
Pair 7	737	0.23	(6 /26632)	786	0.46	(13 /28427)	0.50	−0.23
Pair 8	482	0.06	(1 /17344)	595	0.97	(21 /21637)	0.06	−0.91
Pair 9	653	1.61	(37 /22973)	631	0.57	(13 /22852)	2.82	+1.04
Pair 10	140	2.90	(14 /4824)	107	7.68	(29 /3775)	0.38	−4.78
Total	3,989	2.47	(351 /141903)	4053	4.93	(713 /144694)	weighted paired t-test **p = 0.216**	

Twelve children (1% of acute falciparum malaria) developed severe falciparum malaria; in Dabhine, six severe patients were reported from the NN-villages and two from the ITN-villages. In Myothugyi, one severe patient was reported from the NN-villages and three from the ITN-villages. Thus, overall seven children developed severe malaria in NN villages and five in ITN villages.

**Figure 4:**
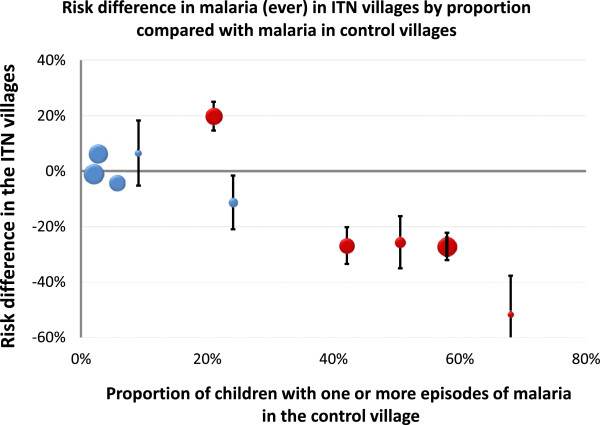
Risk difference in malaria (ever) in ITN villages by proportion compared with malaria in control (NN) villages.

Seven out of 10 village pairs showed a protective efficacy of ITN for both one or more falciparum malaria episodes and one or more vivax malaria episodes while the remaining three village pairs showed the opposite trend. The overall proportion of children having had one or more malaria episode was 16.3% in ITN villages and 22.5% in NN villages, a weighted difference of −6.4% (95% CI; -21.2% to 8.4%), p = 0.35 (Table 3, Figure 4). For *P. falciparum* this was 8.4% (341/4066) in ITN villages and 15.2% (618 /4109) in NN control villages respectively, a weighted difference of −6.9% (95% CI; –18.9% to 5.1%), p = 0.23, and for *P. vivax,* 9.9% (401/4066) in ITN villages versus 12.3% (505/4109) in NN villages, a weighted difference of −2.6% (95% CI; -10.9% to 5.7%), p = 0.25 (Table 3).

**Table 3:** Proportion of children (%) with one or more malaria infections during the study

			***P. falciparum*- ever**				***P. vivax *- ever**				**Any malaria – ever**			
	**ITN (N)**	**Control (N)**	**ITN**	**NN**	**Risk ratio**	**Risk difference**	**ITN**	**NN**	**Risk ratio**	**Risk difference**	**ITN**	**NN**	**Risk ratio**	**Risk difference**
**Dabhine**														
Pair 1	695	740	89	305	0.31	- 28.4	151	243	0.66	- 11.2	214	429	0.53	- 27.2
			(12.8)	(41.2)			(21.7)	(32.9)			(30.8)	(58.0)		
Pair 2	527	662	126	80	1.98	+ 11.8	120	72	2.09	+ 11.9	215	139	1.94	+ 19.8
			(23.9)	(12.1)			(22.8)	(10.9)			(40.8)	(21.0)		
Pair 3	441	285	26	81	0.21	- 22.5	46	64	0.43	- 13.8	67	120	0.36	- 26.9
			(5.9)	(28.4)			(10.4)	(22.5)			(15.2)	(42.1)		
Pair 4	213	176	24	46	0.43	- 14.8	32	60	0.44	- 19.1	53	89	0.49	- 25.7
			(11.3)	(26.1)			(15.0)	(34.1)			(24.9)	(50.6)		
Pair 5	67	72	5	29	0.19	- 32.8	6	33	0.20	- 36.8	11	49	0.24	- 51.7
			(7.5)	(40.3)			(9.0)	(45.8)			(16.4)	(68.1)		
**Myothugyi**														
Pair 6	77	44	8	4	1.14	+ 1.3	6	0	3.35	+ 5.4	12	4	1.71	+ 6.5
			(10.4)	(9.1)			(7.7)	(0)			(15.6)	(9.1)		
Pair 7	745	792	6	13	0.05	- 0.8	1	4	0.20	- 0.4	7	16	0.45	- 1.1
			(0.8)	(1.6)			(0.1)	(0.5)			(0.9)	(2.0)		
Pair 8	489	598	1	23	0.05	- 3.6	6	13	0.56	- 1.0	7	34	0.25	- 4.3
			(0.2)	(3.8)			(1.2)	(2.2)			(1.4)	(5.7)		
Pair 9	664	632	38	13	2.71	+ 3.6	28	6	4.20	+ 3.2	59	17	3.30	+ 6.2
			(5.7)	(2.1)			(4.2)	(1.0)			(8.9)	(2.7)		
Pair 10	148	108	18	24	0.55	- 10.0	5	4	0.92	- 0.3	19	26	0.53	- 11.3
			(12.2)	(22.2)			(3.4)	(3.7)			(12.8)	(24.1)		
Total	4066	4109	341	618			401	505			664	923		
			(8.4)	(15.0)			(9.9)	(12.3)			(16.3)	(22.5)		
Weighted paired t-test			p = 0.23				p = 0.25				p = 0.35			

*P. falciparum*-ever includes *P. falciparum* + mixed, *P. vivax*-ever includes *P. vivax* + mixed. ITN: insecticide treated nets, NN: no nets.

### Age-groups

There was no significant trend for falciparum malaria-infection by age. Restricting the analysis to children under five years of age, similar results were observed compared to the whole study group. Vivax malaria was more common among younger children. Relapse of vivax malaria cannot usually be distinguished reliably from incident infection, although first infections of life are by definition incident. Among children under one year of age, 143 (17,5%) had at least one vivax infection during the 10 month study period (13.7% in ITN villages and 21.0% in NN villages) and children in their second year of age had 185 (19.4%) infections (18.1% in ITN villages and 20.7% in NN villages). After the second year the proportion of children with an episode of vivax malaria decreased steadily by age and only 3.7% of 9-year-old children (3.8% in ITN villages and 3.7% in NN villages) had an episode of vivax malaria in the same period.

### Cross-sectional surveys

Of the 8175 children registered, 7764 (95%) were present at all three ‘prevalence’ surveys. Of the other 411 children, 46 children reportedly died during the study-period (overall child mortality 5.62% in the 42 weeks), (20 from NN villages and 26 from ITN villages; p > 0.2) and 365 had moved out of the area or were absent during one or two of the follow-up surveys (NN 142, ITN 223).

### Malaria prevalence

The prevalences of children with malaria and splenomegaly were measured at the end of the study (January – early February 1999), which coincided with the end of the peak transmission season. A total of 7,828 children were present at this survey. There was considerable variation in malaria prevalence, both for *P. falciparum* and *P. vivax*, and also in the splenomegaly prevalences among the villages (Table 4, and in further detail in Additional files 1, 2, 3 and 4). The overall prevalence of *P. falciparum* was 5.7% (225) in NN villages and 4.2% (161) in ITN villages. Some protective efficacy from impregnated bed nets was seen in six village pairs, while four village pairs showed the opposite effect; overall difference - 1.9% (95% CI; -5.8% to 2.0%), p = 0.30 (Additional files 5, 6 and 7). For *P. vivax* the overall prevalence was 14.2% (564) in NN and 11.6% (446) in ITN villages. Four village pairs suggested protection by ITN while five did not, and in one village pair, both clusters, had no vivax malaria at all; overall difference = −2.8% (95% CI; - 8.5%, 2.9%); p = 0.30. For splenomegaly prevalence, six village pairs suggested protection by ITN, while four did not; overall difference = − 7.3% (95% CI; - 20.1%, 5.5%), p = 0.23. The overall splenomegaly prevalence was 14% in bed net villages and 21% in non-bed net villages.

**Table 4:** ***Plasmodium falciparum, Plasmodium vivax *and splenomegaly prevalences (%) in ITN and control clusters during the peak season at the end of the study (January 1999)**

	**N**	**N**	***P. falciparum***			***P. vivax***			**Spleen**		
	**ITN**	**NN**	**ITN**	**NN**	**Prevalence difference**	**ITN**	**NN**	**Prevalence difference**	**ITN**	**NN**	**Prevalence difference**
**Dabhine**											
Pair 1	659	708	31 (5)	107 (15)	- 10.4	183 (28)	280 (40)	- 11.8	170 (26)	432 (61)	- 35.2
Pair 2	508	644	39 (8)	35 (5)	+ 2.3	120 (24)	135 (21)	+ 2.7	155 (31)	131 (20)	+ 10.2
Pair 3	413	268	17 (4)	21 (8)	- 3.7	57 (14)	83 (31)	- 17.2	63 (15)	90 (34)	- 18.3
Pair 4	203	169	10 (5)	6 (4)	+ 1.4	23 (11)	25 (15)	- 3.5	56 (28)	74 (44)	- 16.2
Pair 5	62	71	3 (5)	7 (10)	- 5.0	23 (37)	22 (31)	+ 7.1	23 (38)	31 (44)	- 5.9
**Myothugyi**											
Pair 6	65	43	14 (22)	3 (7)	+ 14.6	7 (11)	1 (2)	+ 8.4	11 (17)	4 (9)	+ 7.6
Pair 7	717	763	2 (0)	5 (1)	- 0.4	0 (0)	0 (0)	0	6 (1)	23 (3)	- 2.2
Pair 8	462	580	0 (0)	6 (1)	- 1.0	1 (0)	4 (1)	- 0.7	1 (0)	9 (2)	- 1.3
Pair 9	627	620	5 (1)	3 (0)	+ 0.3	9 (1)	2 (0)	+ 1.1	11 (2)	3 (0)	+ 1.3
Pair 10	143	103	40 (28)	32 (31)	- 3.1	23 (16)	12 (12)	+ 4.4	37 (26)	23 (22)	+ 3.5
Total	3859	3969	161 (4)	225 (6)		446 (12)	564 (14)		533 (14)	818 (21)	
Weighted Paired t-test			Pf : -1.9% (−5.8%, 2.0%),			Pv : -2.8% (−8.5%, 2.9%),			Spleen : -7.3% (−20.1%, 5.5%),		
			p-value = 0.30			p-value = 0.30			p-value = 0.23		

*P. falciparum*% and *P. vivax*% indicate the percentage of children with *P. falciparum* or *P. vivax* parasitaemia, both values include mixed infections. ITN: insecticide treated nets, NN: no nets.

### Anaemia and malnutrition

The prevalences of anaemia (Hb <10 g/dL) and malnutrition (Wt/Ht; Z-score < −2) at baseline in the ITN and NN villages were similar. The weighted mean difference in the prevalence of anaemia was −5.2% (ITN-NN; 95% CI: -13.8%, 3.4%) and the weighted mean difference in the prevalence of malnutrition was 0% (ITN-NN; 95% CI: -4%, +4%). The mean haemoglobin level of children during the first cross-sectional survey was lower in Myothugyi (8.85 g/dL; village means varying from 7.84 to 9.24 g/dL) than in Dabhine (9.70 g/dL; village means varying from 9.15 to 10.02 g/dL). The proportion of anaemic children (Hb < 10 g/dL) decreased during the study period in all but two clusters (1 NN and 1 ITN village). At the final cross-sectional survey, the decrease in the proportion of children with anaemia in bed net villages was more pronounced than in control villages in 7 cluster pairs while 3 pairs showed the opposite trend (Table 5). The prevalences of anaemia at the end of the study were not significantly different comparing NN and ITN clusters (weighted mean difference of - 3.3% (95% CI; - 11.3, 4.7), p = 0.38 (Additional file 8).

**Table 5:** Prevalence of anaemia and malnutrition during the 1st and 3rd cross-sectional surveys

**Malnutrition (Wt/Ht < −2Z**^**1**^**)**							
	**ITN clusters**			**NN clusters**			
	**1st prevalence**	**3rd prevalence**	**Change**	**1st prevalence**	**3rd prevalence**	**Change**	**Relative change**
Pair 1	87/692 (13)	67/651 (10)	−2.3%	98/728 (13)	76/701 (11)	−2.6%	−0.3%
Pair 2	61/516 (12)	58/506 (11)	−0.4%	86/651 (13)	102/634 (16)	+2.9%	3.3%
Pair 3	72/435 (17)	71/413 (17)	+0.6%	51/277 (18)	43/266 (16)	−2.5%	−3.1%
Pair 4	62/210 (30)	28/201 (14)	−15.6%	38/169 (22)	32/167 (19)	−3.3%	12.3%
Pair 5	17/64 (27)	6/61 (10)	−16.7%	11/71 (15)	6/70 (9)	−6.9%	9.8%
Pair 6	8/77 (10)	2/65 (3)	−7.3%	3/42 (7)	5/42 (12)	+4.8%	12.1%
Pair 7	105/735 (14)	92/701 (13)	−1.2%	158/780 (20)	92/760 (12)	−8.2%	−7.0%
Pair 8	60/486 (12)	56/450 (12)	+0.01%	67/585 (11)	81/572 (14)	+2.7%	2.7%
Pair 9	87/652 (13)	55/620 (9)	−4.5%	64/627 (10)	78/612 (13)	+2.5%	7.0%
Pair 10	39/148 (26)	8/140 (6)	−20.6%	10/104 (10)	7/100 (7)	−2.6%	18.0%
Total	598/4015 (15)	443/3808 (12)	586/4034 (15)	586/4034 (15)	522/3924 (13)	−1.2%	2.1%
**Anaemia (Hb <10 g/dL)**							
	**ITN clusters**			**NN clusters**			
	**1st prevalence**	**3rd prevalence**	**Change**	**1st prevalence**	**3rd prevalence**	**Change**	**Relative change**
Pair 1	362/695 (52)	299/659 (45)	−6.7%	440/739 (60)	413/708 (58)	−1.2%	5.5%
Pair 2	303/527 (57)	235/508 (46)	−11.2%	332/662 (50)	305/644 (47)	−2.8%	8.4%
Pair 3	228/441 (52)	182/413 (44)	−7.6%	152/285 (53)	125/268 (47)	−6.7%	0.9%
Pair 4	133/213 (62)	88/203 (43)	−19.1%	110/176 (63)	59/169 (35)	−27.6%	- 8.5%
Pair 5	46/67 (69)	22/62 (35)	−33.2%	29/72 (40)	33/71 (46)	+6.2%	39.4%
Pair 6	76/77 (99)	52/65 (80)	−18.7%	42/44 (95)	40/43 (93)	−2.4%	16.3%
Pair 7	485/745 (65)	602/716 (84)	+19.0%	657/792 (83)	558/761 (73)	−9.6%	−28.6%
Pair 8	355/489 (73)	307/461 (67)	−6.0%	532/597 (89)	473/579 (82)	−7.4%	−1.4%
Pair 9	579/664 (87)	443/627 (71)	−16.5%	540/632 (85)	446/619 (72)	−13.4%	3.1%
Pair 10	142/148 (96)	108/143 (76)	−20.4%	104/108 (96)	95/103 (92)	−4.1%	16.3%
Total	2709/4066 (67)	2338/3857 (61)	−6.0%	2938/4107 (72)	2547/3965 (64)	−7.3%	−1.3%

^1^ Weighted paired *t* test 3rd prevalence Wt/Ht - 2 Z score, p-value = 0.094. Relative change = (Prev 3 - Prev 1) NN cluster - (Prev 3 - Prev 1) ITN cluster. ITN: insecticide treated nets, NN: no nets.

The proportion of children with moderate acute malnutrition (Wt/Ht; Z-score < −2) decreased over the study period in 14 of the 20 villages; 8 ITN villages and 6 NN villages (Table 5). At the final nutrition survey the decrease of malnutrition in ITN villages was greater than in control villages in seven cluster pairs, while three pairs showed a greater decrease in control villages. The malnutrition prevalences at the end of the study were not significantly different comparing NN and ITN clusters, either for all children under 10 years (− 1.7% (95% CI; - 3.8, 0.4), p = 0.094), or for the children under five years of age (− 1.8% (95% CI; - 5.9, 2.3), p = 0.35) (Additional file 9).

### Cost-effectiveness analysis of ITN and EDAET

**Figure 5:**
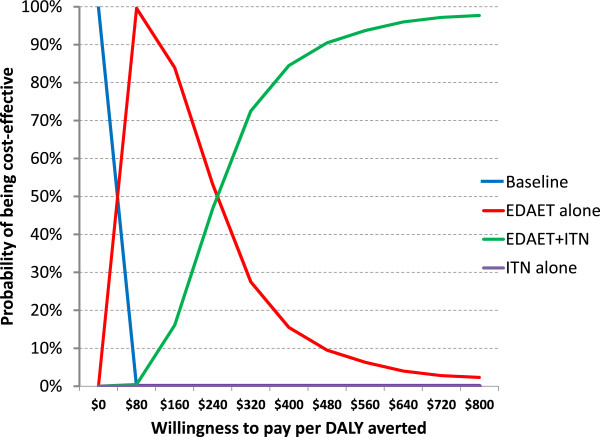
Cost-effectiveness acceptability curves for the four options, accounting for the uncertainty due to the variability in the different study sites and varying levels of willingness to pay per DALY averted.

The average weight of a child under 10 years of age presenting to the malaria programme in Rakhine State was 12.7 kg, which puts the current cost of the drugs for the treatment of falciparum malaria for an average person in this study area at $0.62 and for vivax malaria at approximately $0.3**.** The model output suggests that in the absence of ITN or EDAET, 1.4 DALYs are accumulated per 1,000 person-weeks at no cost to the provider. The use of ITN would add a cost of $35 and reduce the number of DALYs to 0.69, or an incremental cost-effectiveness ratio (ICER) of $51/DALY averted. The use of EDAET alone would cost $23 per 1000 person-weeks and reduce DALYs to 0.19, or an ICER of $19 per DALY averted (i.e. nearly three times less). While both options are considered cost-effective, the implementation of ITN *instead of* EDAET would incur higher costs and avert less than a third of the number of DALYs. Where EDAET is already in place the introduction of ITN would avert an additional 0.2 DALYs at a cost of $148 per DALY averted. The cost-effectiveness acceptability curves (Figure 5) illustrate these results, and show that if policy makers are willing to pay over approximately $280 per DALY averted, the combination of both ITN and EDAET is likely to be cost-effective. If resources are more constrained then EDAET alone will always dominate the use of ITN alone.

This analysis derives from the data in this study and, therefore, specifically to children in this area of Western Myanmar. EDAET is likely to be equally effective in adults, but ITNs are likely to be less effective in the adult population who are often outside dwellings in the early evening, and go to sleep later and leave the home earlier than children. Thus the comparative economic advantage of EDAET over ITN is likely to be even greater in adults and, therefore, the overall advantage of EDAET over ITN for the population overall is predicted to be greater than reported here.

This analysis assumes no impact of EDAET on incidence, although a comparison of the baseline and post-intervention surveys in the control villages suggests that EDAET might itself be reducing transmission. This assumption does not impact on the incremental cost/gain of either of the strategies compared with each other, but it could potentially underestimate the relative advantage of EDAET as compared with a baseline of doing nothing, while overestimating that of ITN.

## Discussion

The results of this study suggest that ITNs provided some protection for children against malaria in most villages in this area of Rakhine State, but they did not in the others, and the overall result was that there was no significant benefit evident from their deployment. Malaria episodes were less common overall in villages with ITN than in the control group, because differences in favour of ITN were greater than differences in favour of no nets (Figures 3 and 4), but when these data were aggregated per cluster, systematic use of ITN was not found to reduce either falciparum or vivax malaria significantly. Importantly they also did not attenuate the adverse consequences of malaria on anaemia or growth. This study focussed on children, as they are the most vulnerable age group for malaria in this area. This provided the highest estimate of potential protective efficacy and thus benefit from ITN, as children go to bed earlier and they sleep longer than adults (particularly relevant in this area with early evening outdoor biting vectors [22]), and they are less likely to travel far from the house. As all villagers in the ITN clusters were provided with a net, any positive outcome could have been further enhanced through a mass insecticidal effect on the malaria vectors. In these villages malaria affected children more than adults but malaria in the South East Asian region is often a disease particularly affecting young mobile adults who work in the forest and do not tend to use nets, so any benefits demonstrated here are likely to be greater than in the total population at risk in the region [23-25]. Adherence with instructions for correct use of ITN was generally good, but as the benefits were small, incorrect use did not lead to more malaria. Even though the ITN and control clusters were selected randomly, the malaria prevalence before the study was higher in the study villages than in the control villages. This might indicate a higher endemicity, which could have influenced the incidence and final prevalence results of the ITN clusters, but transmission intensity varies greatly per year and the differences were small and unlikely to make a material difference to the results. This study was tightly controlled, with a high coverage and user rate compared to the normal context of use. Despite this no significant overall benefit from ITN deployment in terms of malaria protection could be demonstrated. The proportion of children with anaemia decreased both in villages provided with ITNs and in the control villages over the duration of the study. This is probably largely because diagnosis and effective treatment of malaria were provided in all villages, and because children with anaemia were treated promptly, which emphasizes the benefit provided by prompt and effective anti-malarial treatment in such areas of low unstable transmission [5,26].

The most likely explanation for this rather disappointing result from ITN deployment in this malaria endemic area of Western Myanmar is the early evening biting pattern and strong preference for outdoor biting of most malaria vectors in this area [22], as in many other areas of South-East Asia [10-15,27-30]. Focussing on the human bite catches between July 1998 and January 2000 (n = 2,895), the overall peak biting time was between 6 pm and 7 pm, and over half of the anophelines (51%) were caught before 8 pm. This would differentiate any benefits of ITNs between children, who are often in or near an ITN at this time, and adults who often are not. As the behaviour of malaria vectors differs so much, particularly in Asia, entomological information is essential before ITNs are deployed. These somewhat negative results contrast with the widespread positive perception of the uniform value of ITNs in malaria control. A systematic review of ITN evaluations [9], which included 22 studies from Africa, Asia and South America, concluded that ITNs reduce childhood morbidity and mortality and that ITNs should be employed in all malarious areas. Over the past decade ITNs have been taken up enthusiastically as a key component of many malaria control programmes, but caution is needed in interpreting the findings from bed net studies with different designs and analytical methods. According to Lengeler, of 29 identified studies conducted in Asia, only four studies [10,12,31,32] used correct statistical procedures for their trials and were included in the meta-analysis. Another study [11] did use correct statistical procedures, but was not included because it studied only pregnant women. Of these five “statistically correct” studies, three showed reduced morbidity [10,12,31] while the other two did not [11,32]. In other studies, non-comparable control groups were used or ITN were allocated at a village-level, whereas end-points were calculated for individuals. There is no doubt concerning the consistent and large benefits provided by ITNs in Africa which fully justifies current deployment initiatives. In contrast, the epidemiology of malaria in Asia is extremely heterogeneous. Transmission is highly seasonal and unstable and intensities vary greatly over short distances, and also show great variation from year to year. It is not surprising that ITNs are relatively ineffective in areas of unstable malaria where the principal malaria vectors bite outdoors early in the evening, before people go in or near their ITN. This and previous studies argue against generalising from ITN studies in Africa to the rest of the malaria affected world. Where the main malaria vectors bite mainly indoors after adults and children have gone to sleep, ITNs should be very effective, but in many places, this is not the case. This is not to say that ITNs provide no benefit in these places– they do protect against malaria [33], albeit sometimes to a small extent, and if cost were no obstacle then everyone in malaria endemic areas should certainly be provided with an ITN. But funds for malaria control are usually limited and ITN deployment is often included uncritically in many malaria control efforts in Asia in preference to other control measures.

Early diagnosis and effective treatment reduces malaria morbidity and mortality, but in a large proportion of patients in south and Southeast Asia, the diagnosis of malaria is still made clinically and, therefore, incorrectly. A lack of resources means that most patients with malaria in Myanmar and in adjacent countries under similar constraints still receive inadequate treatment. Many patients take a few tablets of artesunate until they feel better. Sometimes chloroquine is still used which is inexpensive but ineffective. Artesunate-based combination treatment is a proven highly effective treatment, which significantly reduces malaria transmission in low-transmission areas. Without accounting for these transmission blocking properties, the present economic model indicates that effective diagnostic and treatment (EDAET) services are less costly and substantially more effective than ITN, when comparing each of these to a baseline of no intervention, which is still the reality in some areas in Myanmar. Once EDAET is implemented effectively, ITNs can still provide additional benefit.

During the extended interval since this study was carried out there has been a consistent and substantial decline in malaria incidence across the region, including many areas in Myanmar even when allowing for improved reporting rates [34]. While the Rakhine state still accounts for approximately half the burden in Myanmar, the incidence rates reported in this study are likely to be higher than those that would be documented today in this region. This could further strengthen the argument in favour of EDAET. Lower incidence of malaria will imply that protective measures which require the same costs applied to the entire population will be relatively less cost-effective than those that target infected individuals such as diagnosis and treatment.

ITNs are very popular with international donor agencies. Millions of dollars are spent on ITN programmes in this populous malaria endemic region. ITNs are both provided free by donor organizations or promoted through social marketing, encouraging the population to purchase their own ITN. In this malaria endemic region of Myanmar, the economic modelling based on the study data and contemporary prices suggests that early-diagnosis and effective treatment is substantially more cost-effective than deployment of LLINs as a malaria control measure. This argues for careful evaluation in each region before large-scale deployment. The findings here provide some evidence of a protective efficacy of ITNs in areas of higher endemicity within this region, suggesting that they can be a cost-effective intervention in the context of improving child health. If, however, donor funding is targeting specifically the control of malaria and ultimately its elimination from the region it seems likely that improving preventive, diagnostic and curative interventions for all ages and particularly in adult, often migrant males, could offer better returns on investment.

## Conclusion

Malaria infections, palpable splenomegaly, haemoglobin concentrations, and weight for height, did not differ significantly during the study period between villages with and without ITNs. The limited efficacy of ITNs may be explained by the biting behaviour (peak biting time between 1800 and 1900 hours, mainly outdoors) of the most common *Anopheles* mosquito vectors [22]. Given the lack of significant efficacy and relatively high costs of ITNs, the first priority in implementation of malaria control interventions in this area should be the provision of effective diagnosis and treatment. Where EDAET services are already in place and sufficient budgets are available then the use of ITN can be cost-effective.

## Competing interests

The authors have no conflicts of interest concerning the work reported in this paper.

## Authors’ contributions

FS, JAS and NJW conceived the idea for the paper. JAS, KS, MKK and FS conducted the analysis. YL conducted the economic evaluation. FS and NJW wrote the paper with input from all other authors. All authors have approved the final version of this manuscript.

## Supplementary Material

Additional file 1 **Change in prevalence of falciparum malaria, vivax malaria, and spleen enlargement from before to after the intervention.** Table showing change in prevalence of falciparum malaria, vivax malaria, and spleen enlargement comparing the prevalence before (December 1997) and after (January 1999) the intervention and the relative change between ITN and NN villages Click here for file

Additional file 2 **Prevalence of falciparum malaria before the study.** Description Prevalence of falciparum malaria before the start of the study comparing ITN and NN villages. Click here for file

Additional file 3 **Prevalence of vivax malaria before the study.** Description Prevalence of vivax malaria before the start of the study comparing ITN and NN villages. Click here for file

Additional file 4 **Prevalence of splenomegaly before the study.** Description Prevalence of palpable spleens before the start of the study comparing ITN and NN villages. Click here for file

Additional file 5 **Prevalence of falciparum malaria after the study.** Description Prevalence of falciparum malaria after the study comparing ITN and NN villages. Click here for file

Additional file 6 **Prevalence of vivax malaria after the study.** Description Prevalence of vivax malaria after the study comparing ITN and NN villages. Click here for file

Additional file 7 **Prevalence of splenomegaly after the study.** Description Prevalence of palpable spleens after the study comparing ITN and NN villages. Click here for file

Additional file 8 **Effects on anaemia.** Description Change of proportions of children with anaemia (Hb < 10 g/dL) from the 1st to the 3rd cross-sectional survey in ITN and NN villages. Click here for file

Additional file 9 **Effects on malnutrition.** Description Change of proportions of children with malnutrition (Wt/Ht < −2Z) from the 1st to the 3rd cross-sectional survey in ITN and NN villages. Click here for file
