# Supplementary material for: The effect of insecticide-treated bed nets on the incidence and prevalence of malaria in children in an area of unstable seasonal transmission in western Myanmar
Source: Malar J. 2013 Oct 11;12:363. doi: 10.1186/1475-2875-12-363 (PMC3854704; doi:10.1186/1475-2875-12-363)
Supplement: Additional file 1 — Change in prevalence of falciparum malaria, vivax malaria, and spleen enlargement from before to after the intervention. Table showing change in prevalence of falciparum malaria, vivax malaria, and spleen enlargement comparing the prevalence before (December 1997) and after (January 1999) the intervention and the relative change between ITN and NN villages [file 1475-2875-12-363-S1.docx]

**Additional file 1.** Change in prevalence of falciparum malaria, vivax malaria, and spleen enlargement comparing the prevalence before (December 1997) and after (January 1999) the intervention and the relative change between ITN and NN villages**.**

|  | Changes in prevalence (%) of falciparum malaria | | | Changes in prevalence (%) of vivax malaria | | | Changes in prevalence (%) of spleen enlargement | | |
| --- | --- | --- | --- | --- | --- | --- | --- | --- | --- |
|  | ITN villages | NN villages | relative change | ITN villages | NN villages | relative change | ITN villages | NN villages | relative change |
| Pair 1 | 25 | 22 | 3 | 0 | - 8 | 8 | 37 | 7 | 30 |
| Pair 2 | 21 | 14 | 7 | 13 | - 6 | 19 | 22 | 14 | 8 |
| Pair 3 | 18 | 14 | 4 | 13 | - 19 | 32 | 27 | 27 | 0 |
| Pair 4 | 16 | 9 | 7 | 13 | 35 | - 22 | 0 | 15 | - 15 |
| Pair 5 | 22 | 43 | - 21 | - 15 | - 24 | 9 | - 10 | - 4 | - 6 |
| Pair 6 | - 3 | 13 | - 16 | - 5 | - 2 | - 3 | 21 | - 2 | 23 |
| Pair 7 | 1 | - 1 | 2 | 0 | 0 | 0 | 15 | 26 | - 11 |
| Pair 8 | 0 | 2 | - 2 | 2 | 0 | 2 | 24 | 7 | 17 |
| Pair 9 | 6 | 1 | 5 | 6 | 0 | 6 | 10 | 1 | 9 |
| Pair 10 | 12 | 4 | 8 | - 8 | - 7 | - 1 | 6 | - 2 | 8 |
