# Supplementary figures and images for: The effect of insecticide-treated bed nets on the incidence and prevalence of malaria in children in an area of unstable seasonal transmission in western Myanmar
Source: Malar J. 2013 Oct 11;12:363. doi: 10.1186/1475-2875-12-363 (PMC3854704; doi:10.1186/1475-2875-12-363)

## Slide 1
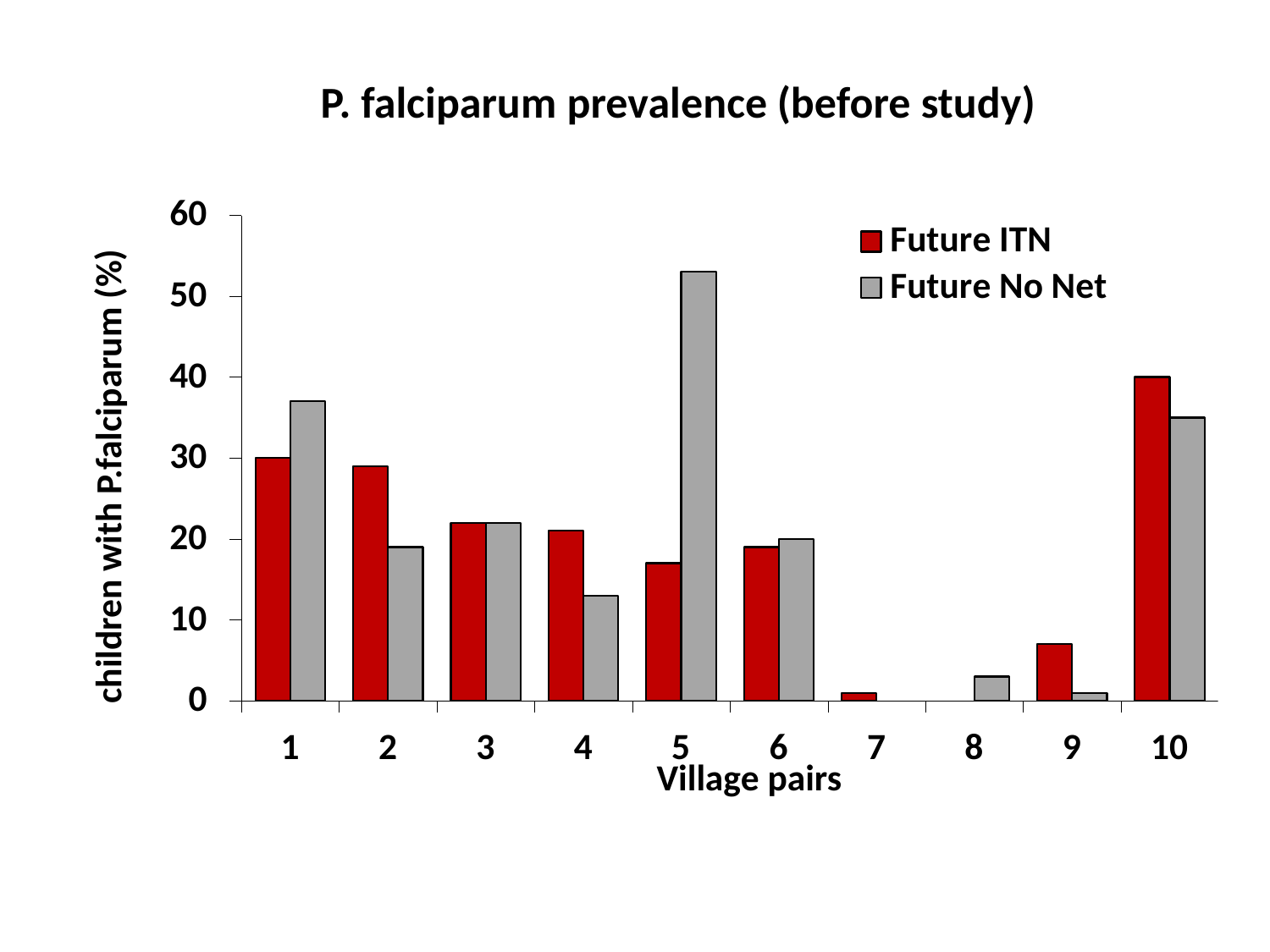

Supplement: Additional file 2 — Prevalence of falciparum malaria before the study. Description Prevalence of falciparum malaria before the start of the study comparing ITN and NN villages. [file 1475-2875-12-363-S2.pptx]

## Slide 1
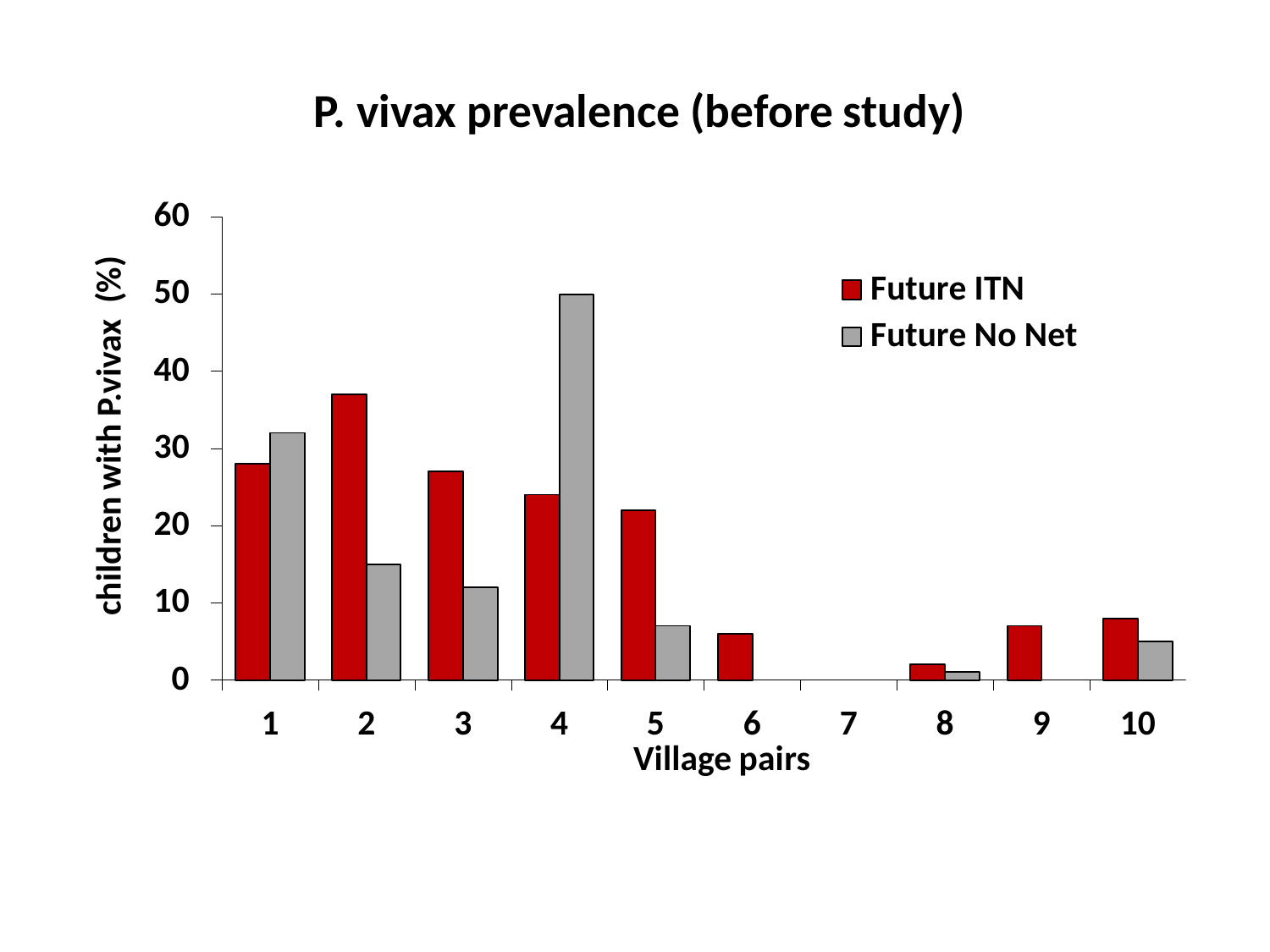

Supplement: Additional file 3 — Prevalence of vivax malaria before the study. Description Prevalence of vivax malaria before the start of the study comparing ITN and NN villages. [file 1475-2875-12-363-S3.pptx]

## Slide 1
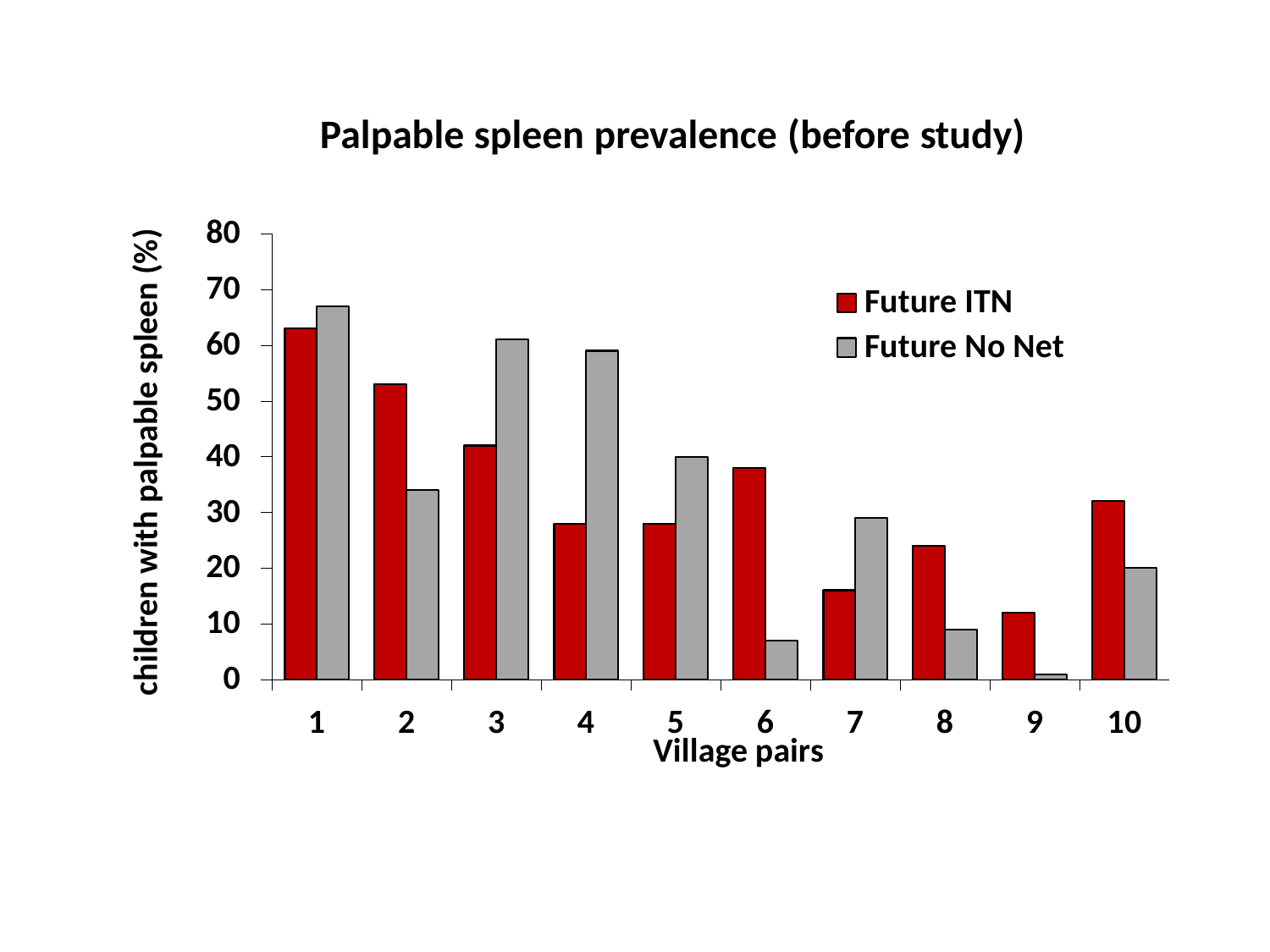

Supplement: Additional file 4 — Prevalence of splenomegaly before the study. Description Prevalence of palpable spleens before the start of the study comparing ITN and NN villages. [file 1475-2875-12-363-S4.pptx]

## Slide 1
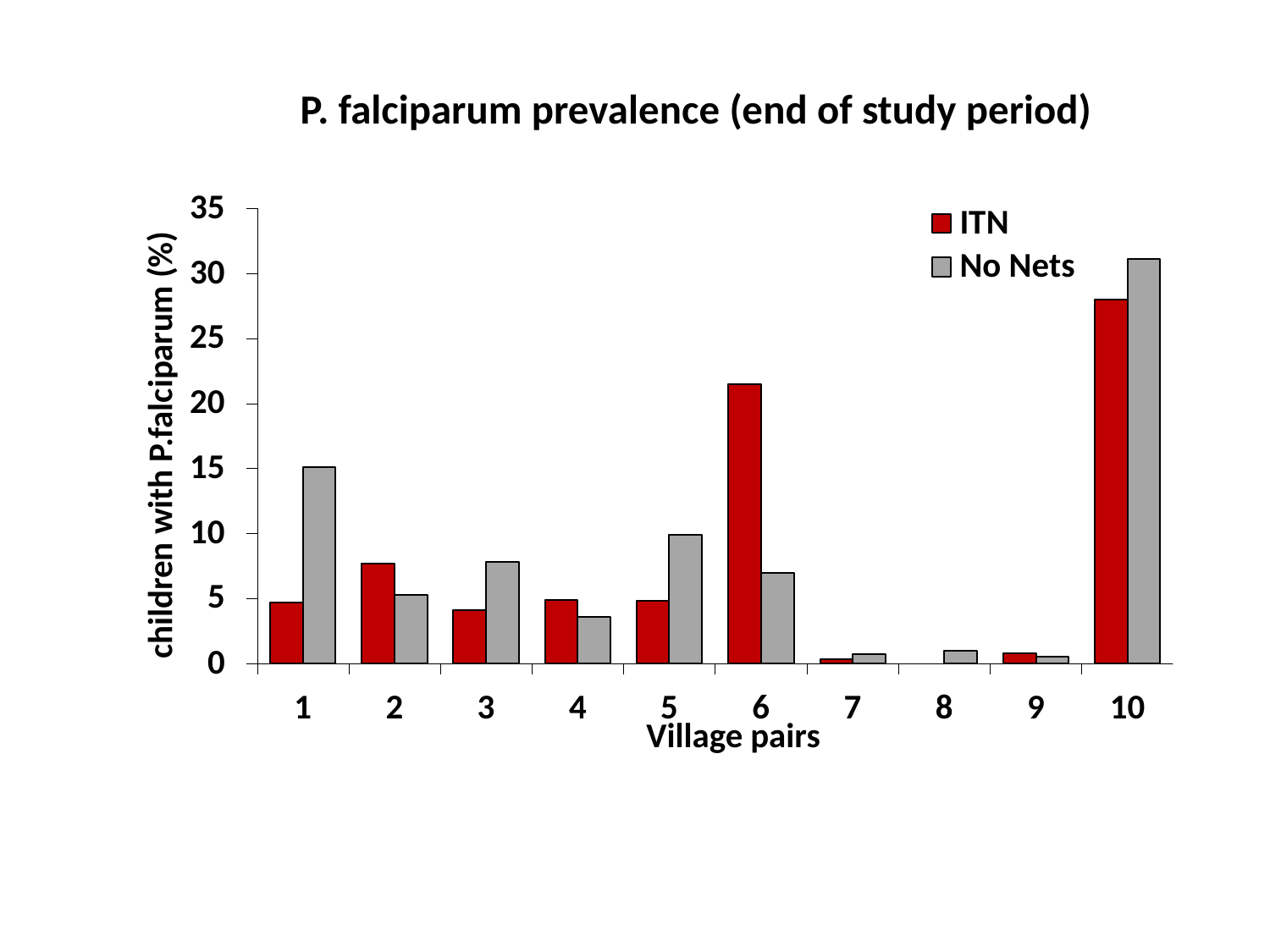

Supplement: Additional file 5 — Prevalence of falciparum malaria after the study. Description Prevalence of falciparum malaria after the study comparing ITN and NN villages. [file 1475-2875-12-363-S5.pptx]

## Slide 1
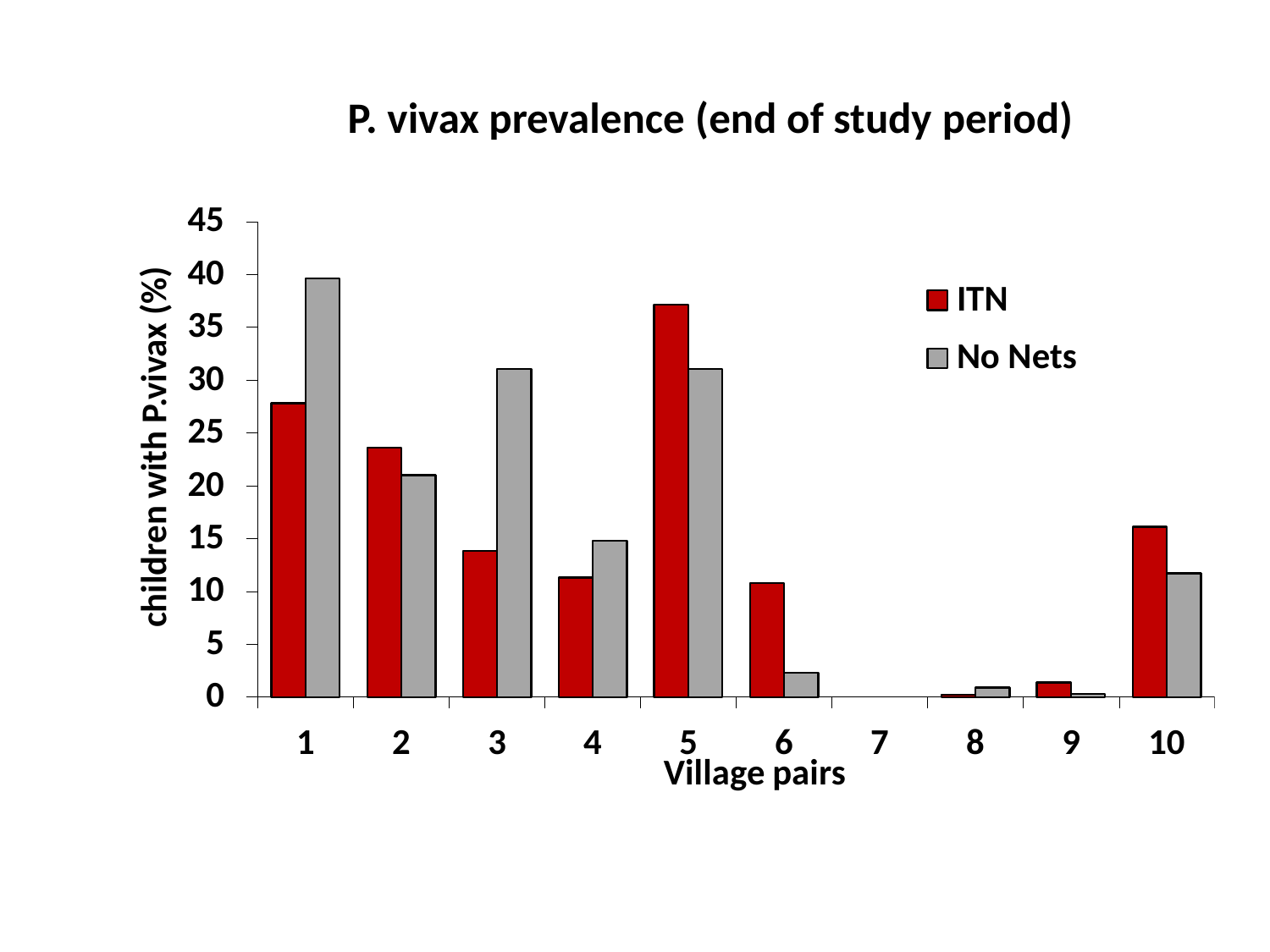

Supplement: Additional file 6 — Prevalence of vivax malaria after the study. Description Prevalence of vivax malaria after the study comparing ITN and NN villages. [file 1475-2875-12-363-S6.pptx]

## Slide 1
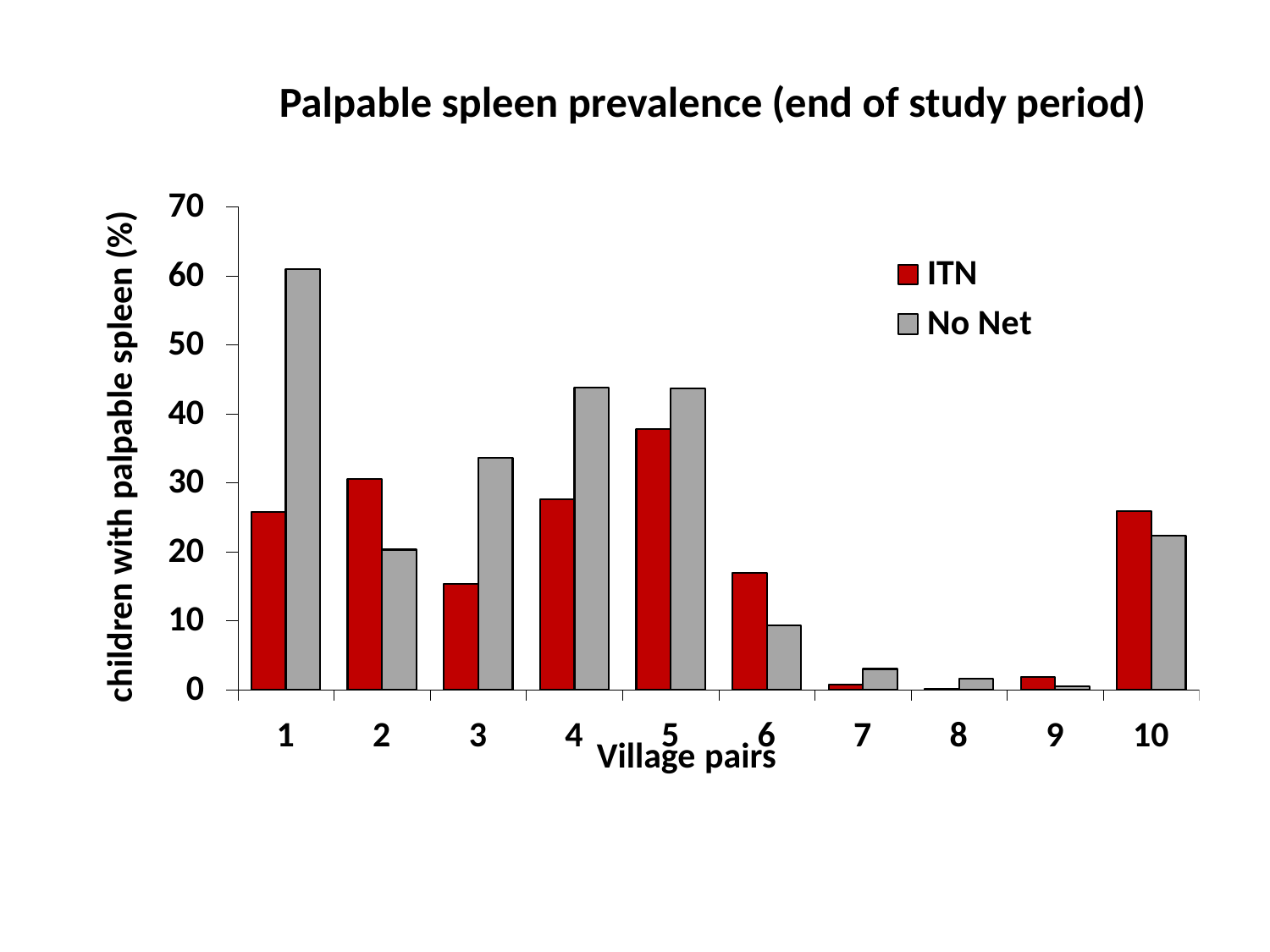

Supplement: Additional file 7 — Prevalence of splenomegaly after the study. Description Prevalence of palpable spleens after the study comparing ITN and NN villages. [file 1475-2875-12-363-S7.pptx]

## Slide 1
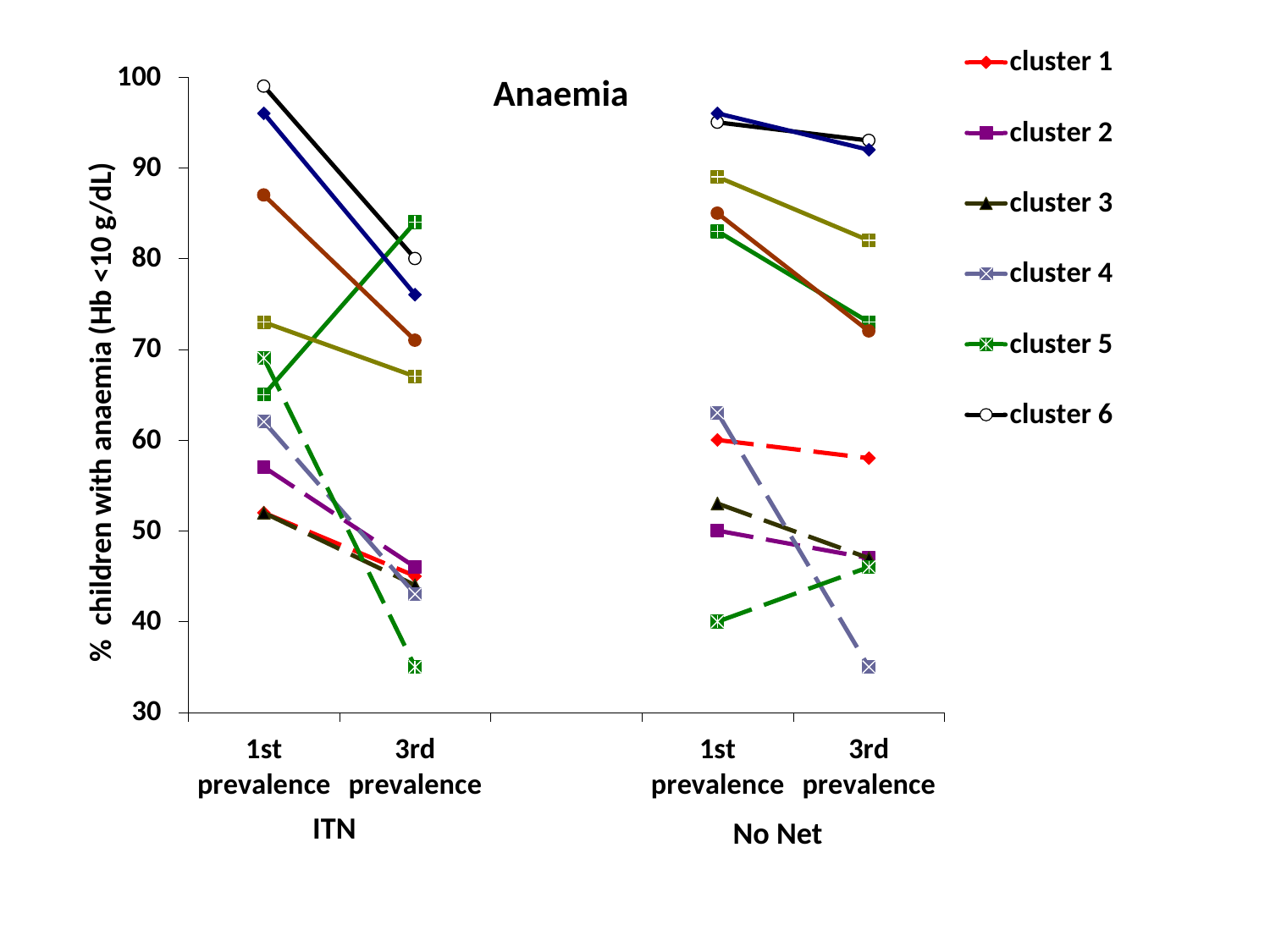

Supplement: Additional file 8 — Effects on anaemia. Description Change of proportions of children with anaemia (Hb < 10 g/dL) from the 1st to the 3rd cross-sectional survey in ITN and NN villages. [file 1475-2875-12-363-S8.pptx]

## Slide 1
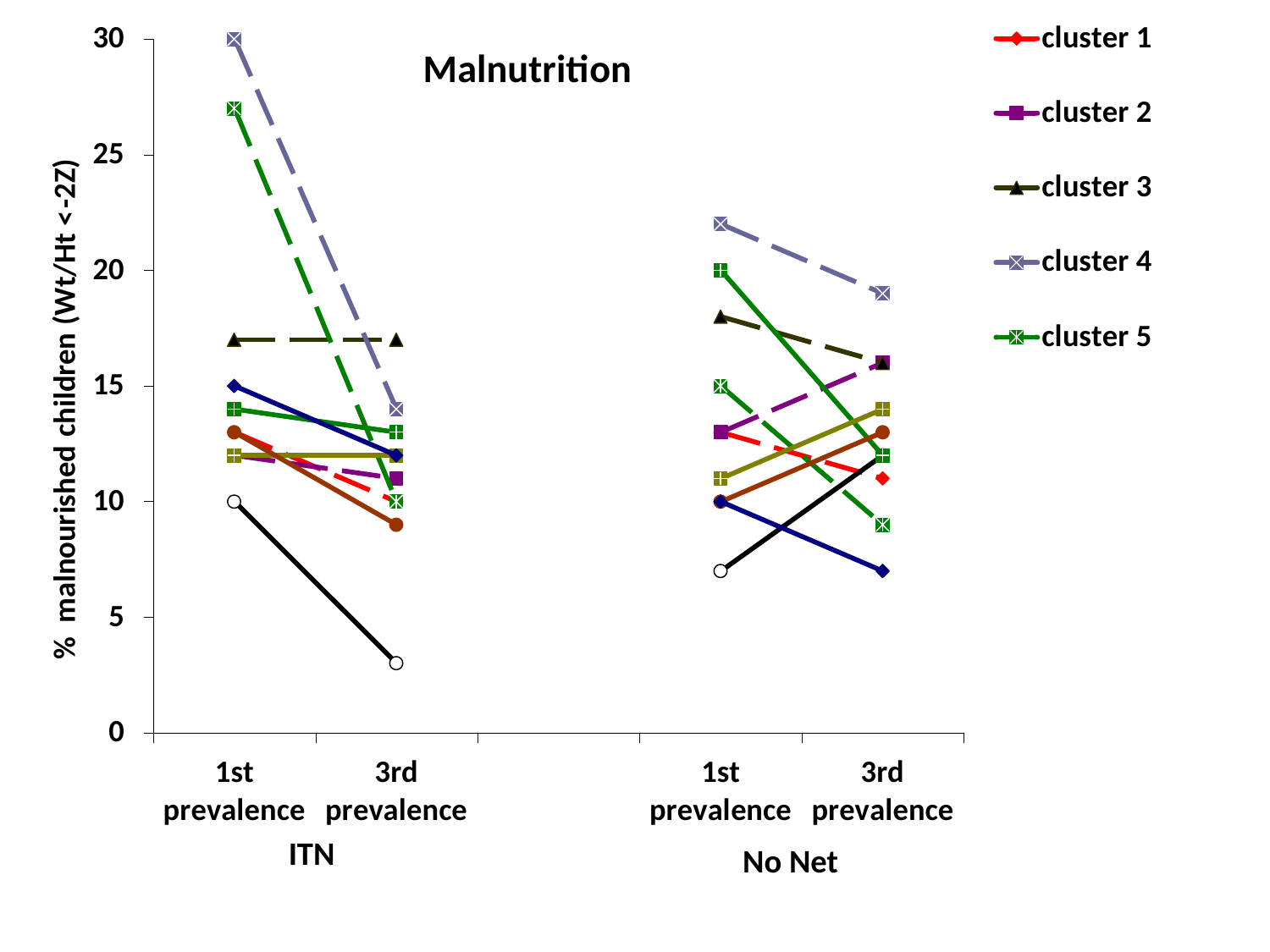

Supplement: Additional file 9 — Effects on malnutrition. Description Change of proportions of children with malnutrition (Wt/Ht < −2Z) from the 1st to the 3rd cross-sectional survey in ITN and NN villages. [file 1475-2875-12-363-S9.pptx]
